# Trends in Hearing Aid Use Among Older Adults in the United States, 2011-2018

**DOI:** 10.1093/geroni/igaa057.346

**Published:** 2020-12-16

**Authors:** Nicholas Reed, Emmanuel Garcia Morales, Amber Willink

**Affiliations:** 1 Johns Hopkins University, Baltimore, Maryland, United States; 2 Johns Hopkins Bloomberg School of Public Health, Baltimore, Maryland, United States; 3 The University of Sydney, Sydney, New South Wales, Australia

## Abstract

Hearing loss among older adults is prevalent and associated with dementia and health care utilization. However, cross-sectional data suggest less than 20% of adults with hearing loss use hearing aids. There is a paucity of studies examining trends in hearing aid ownership over time. This study analyzed data from the 2011, 2015, and 2018 cycles of the National Health Aging and Trends Study (NHATS), a nationally-representative longitudinal study of Medicare Beneficiaries. Participants were asked “in the last month, [have you/has [he/she]] use a hearing aid or other hearing device?” (“yes” or “no”). Among a weighted sample of Medicare Beneficiaries 70 years and older (26.47 million in 2011; 29.70 million in 2015; and 33.28 in 2018), the overall proportion who own and use hearing aids rose from 14.96% in 2011 to 16.90% in 2015 to 18.45% in 2018. As age increased so did the proportion of older adults who used hearing aids. A smaller proportion of Black Americans used hearing aids across time and experienced a smaller overall increase in the proportion in hearing aid ownership over the 8-year period compared to White Americans (+0.78% vs. +4.30%). Black women had the lowest rates of hearing aids use across the 8-year period. Notably, older adults at less than 100% of the federal poverty level experienced an overall decrease in proportion of hearing aid ownership and use. This study lays the groundwork to examine the impact of the Over-the-Counter Hearing Aid Act of 2017 across subpopulations when it takes effect in 2021.

